# Safe recovery after cesarean in rural Africa: Technical consensus guidelines for post‐discharge care

**DOI:** 10.1002/ijgo.14284

**Published:** 2022-06-25

**Authors:** Fredrick Kateera, Bethany Hedt‐Gauthier, Amy Luo, Anne Niyigena, Grace Galvin, Sadoscar Hakizimana, Rose L. Molina, Adeline A. Boatin, Prisca Kasonde, Juliet Musabeyezu, Joseph Ngonzi, Robert Riviello, Katherine Semrau, Félix Sayinzoga

**Affiliations:** ^1^ Partners In Health/Rwanda Kigali Rwanda; ^2^ Department of Global Health and Social Medicine Harvard Medical School Boston Massachusetts USA; ^3^ Program in Global Surgery and Social Change Harvard Medical School Boston Massachusetts USA; ^4^ Department of Global Health and Population Harvard T.H. Chan School of Public Health Boston Massachusetts USA; ^5^ Ariadne Labs, Brigham and Women's Hospital Harvard T.H. Chan School of Public Health Boston Massachusetts USA; ^6^ Department of Obstetrics and Gynecology Beth Israel Deaconess Medical Center Boston Massachusetts USA; ^7^ Department of Obstetrics and Gynecology Massachusetts General Hospital Boston Massachusetts USA; ^8^ Obstetrics and Gynecology Consultant Lusaka Zambia; ^9^ Harvard Medical School Boston Massachusetts USA; ^10^ Department of Obstetrics and Gynecology Mbarara University of Science and Technology Mbarara Uganda; ^11^ Center for Surgery and Public Health Brigham and Women's Hospital Boston Massachusetts USA; ^12^ Department of Medicine Harvard Medical School Boston Massachusetts USA; ^13^ Division of Global Health Equity Brigham and Women's Hospital Boston Massachusetts USA; ^14^ Maternal, Child and Community Health Division Rwanda Biomedical Center Kigali Rwanda

**Keywords:** global surgery, maternal health, postpartum, Rwanda, sub‐Saharan Africa

## Abstract

Despite increasing cesarean rates in Africa, there remain extensive gaps in the standard provision of care after cesarean birth. We present recommendations for discharge instructions to be provided to women following cesarean delivery in Rwanda, particularly rural Rwanda, and with consideration of adaptable guidelines for sub‐Saharan Africa, to support recovery during the postpartum period. These guidelines were developed by a Technical Advisory Group comprised of clinical, program, policy, and research experts with extensive knowledge of cesarean care in Africa. The final instructions delineate between normal and abnormal recovery symptoms and advise when to seek care. The instructions align with global postpartum care guidelines, with additional emphasis on care practices more common in the region and address barriers that women delivering via cesarean may encounter in Africa. The recommended timeline of postpartum visits and visit activities reflect the World Health Organization protocols and provide additional activities to support women who give birth via cesarean. These guidelines aim to standardize communication with women at the time of discharge after cesarean birth in Africa, with the goal of improved confidence and clinical outcomes among these individuals.

## Synopsis

Recommendations of a Technical Advisory Group for postpartum instructions for women after cesarean birth in rural Africa.

## 1 INTRODUCTION

During the postpartum period, women face an array of physical, social, and psychological changes. Care during this time strongly contributes to long‐term health and well‐being for the woman and her infant.^1^ The World Health Organization (WHO) provides guidelines for postpartum care for mothers and infants with a special focus on low‐ and middle‐income countries (LMICs).^2^ These guidelines address the timing, location, content, and number of postpartum contacts and include recommendations on maternal and newborn health care. Similarly, the American College of Obstetricians and Gynecologists National Institute for Health and Clinical Excellence, and Save the Children have published clinical practice guidelines on routine postpartum care for the mother‐infant dyad.^3^, ^4^, ^5^

Women who give birth by cesarean face additional unique challenges, with significantly higher risk of postpartum complications, such as infection and hemorrhage, compared with women who have a vaginal birth.^6^ Factors such as wound healing, functional recovery, and reproductive life planning require specific considerations beyond guidelines for women delivering vaginally. Given the rising cesarean rates globally, cesarean‐specific postpartum care guidelines are crucial for the full recovery of these women.^7^ However, the aforementioned guidelines do not include recommendations specific to women who deliver via cesarean, and, based on our research, no such postpartum instructions currently exist in the published literature.

In sub‐Saharan Africa (SSA), cesarean births have increased over three‐fold since the 1990s.^7^ For women giving birth via cesarean, barriers to seeking postpartum care in these settings include financial constraints, transportation costs, conflicting or incomplete information, and misperceptions around the importance of postpartum care.^8^ Across several studies, women delivering via cesarean in Rwanda reported a lack of instructions or conflicting instructions at the time of discharge.^8^, ^9^, ^10^ A scoping review found that very few studies based in SSA comment on cesarean recovery activities or postoperative care pathways after hospital discharge, and even fewer studies provide concrete recommendations.^11^ Here, we outline recommendations for discharge instructions to be provided to women who have given birth by cesarean in SSA to optimize their recovery. These recommendations were developed through expert consensus with a focus on rural Rwanda and discussions on how these instructions could be generalized to other SSA contexts.

## 2 MATERIALS AND METHODS

### 2.1 Technical advisory group convening

These consensus guidelines were developed by a Technical Advisory Group (TAG), individuals convened by the TAG chair (FK) and study principal investigator (BHG) with the goal of identifying the most pressing needs in post‐cesarean care and to develop specific guidelines to address those gaps. The TAG included members with expertise in cesarean birth and maternal health care in Rwanda, as the primary goal was to develop instructions to be given to women in rural Rwanda. Additional experts in maternal health working in other SSA countries were invited with the goal of increasing the relevance of these recommendations to other contexts. The TAG included experts representing a range of work in health ministries, non‐governmental organizations, clinical care, and academia, with expertise in obstetrics and gynecology; maternal, newborn, and child care; community health; health system strengthening; implementation science; and surgical care from Rwanda, Uganda, Zambia, and the USA.

Over the course of 4 months, all TAG members attended three meetings; all meetings were hosted virtually on the Zoom platform. In the first meeting, hosted February 25, 2021, the TAG prioritized key areas of focus based on findings from a scoping review and their professional experience. The three recommended areas of focus were: general postpartum care, wound care, and mental health. Between the first and second meetings, the TAG members joined one of three subgroups each aligned to an area of focus. For the assigned topic area, each subgroup developed the first draft of instructions to be provided to the mother at discharge and recommendations for formal follow‐up visits with the health sector, including the visit timing and visit content. Before the second meeting, each TAG member provided input on the sets of recommendations drafted by the two other subgroups.

The second TAG meeting, hosted June 1, 2021, focused on proposed draft recommendations and discussed edits and comments received from the TAG members. After this meeting, a compiled draft of recommendations was circulated to the full TAG. In the last meeting, hosted September 23, 2021, TAG members made final additions and recommendations for the consensus guidelines and discussed ways to consolidate the follow‐up visit schedule to minimize the financial and physical burden of follow up. All members reviewed the compiled instructions and provided feedback on any remaining areas under discussion. Final instructions shared in this manuscript were approved by all TAG members after reaching group consensus.

### 2.2 Ethical considerations

This paper was generated through consensus of a technical advisory group; therefore, no ethics approval was required.

## 3 RESULTS

### 3.1 Instructions to be provided to women after cesarean at the time of discharge

The full set of discharge instructions are provided in Tables 1 and 2. Here, we summarize some additional considerations discussed by the TAG. These instructions were developed assuming that most women are discharged on postoperative day 3, which is typical in Rwanda and other SSA contexts unless there is a clinical indication necessitating an extended facility stay.^12^, ^13^ The TAG members include additional guidelines in Table 3 for those who experience complications and note that individualized guidelines are still needed.

**TABLE 1:** Normal and abnormal recovery symptoms during recovery from cesarean birth

Category	Symptoms
Normal recovery	There are a wide range of symptoms that you will experience as you recover from your cesarean delivery. Here we summarize a few that are normal after delivery. It is normal to have vaginal bleeding and uterine cramps that resolve within 2 weeks of delivery. Expect bleeding to decrease within the 2 weeks post‐delivery. Vaginal spotting will change in color, from bright red, dark red, brown, yellow, to clear before it stops. Cramps may be worse when you are breastfeeding. It is also normal to have a small amount of clear or pinkish/reddish‐brown discharge from your wound for a few days after discharge. It is also normal for your breasts to swell and feel sore 1–2 weeks after delivery. It is normal to feel stressed and emotional after birth. You may: feel anxious, irritable, or tearful; be in low spirits; be unable to enjoy things you normally enjoy; have a low level of energy; have poor sleep; have difficulty concentrating on tasks. However, if these symptoms continue for longer than 2 weeks, please seek help from a healthcare worker as symptoms may represent postpartum depression
Abnormal recovery	The following are not expected during normal recovery; if you experience any of the following, please contact a healthcare provider as soon as possible. Seek care right away (within 24 h): Pain in your chest, racing or fluttering heartbeat in your chest. Abdominal pain or pain at the wound site that is extreme, even with medication, or if the pain gets worse. Difficulty breathing or shortness of breath. Seizures. Symptoms of seizures include one of the following: loss of consciousness or confusion, falling for no apparent reason, uncontrollable movement (jerking movement of arms or legs, convulsions, or trembling/shivering), rapid blinking, inability to break a fixed gaze, numbness or prickling sensation, loss of bladder control. Excessive vaginal bleeding (soaking through one pad per hour for 2 h) or blood clots the size of an egg or bigger, abdominal pain and/or foul smelling or yellow‐green vaginal discharge. Red or swollen leg that is painful or warm to touch, calf pain. Fever (hot forehead, flushed cheeks, and body weakness and sweating) with or without shivering. Headache that does not improve, headache accompanied by one or more of the symptoms of visual disturbances (blurred vision, partial blindness, or any changes to your vision), nausea, vomiting, upper or lower abdominal pain outside the navel region, feeling faint, convulsions (in the first few days after birth). Persistent nausea or vomiting. Dizziness. Persistent thick yellow/white or bloody discharge from wound, redness around incision, induration around the incision, or separation of incision (gaping). Breasts feel painful (burning sensation or very tender) and fever. Thoughts about harming self or baby. Violence at home, feeling unsafe at home. Seek care urgently (within 1–3 days): Not having used the toilet or straining to pass stools for more than 4 days. Inability to pass gas for more than 24 hours and experiencing bloating or swelling in the belly area. Bleeding hemorrhoids. Feeling in low spirits, tearful, unable to enjoy things you normally enjoy, having low energy, poor sleep, and difficulty concentrating on tasks for longer than 2 weeks (14 days) after childbirth. Inability to care for yourself or the baby. Severe anxiety, agitation, or panic. Repetitive or persistent thoughts that are unwanted and cause distress.

**TABLE 2:** Core postpartum instructions to be communicated to all women post‐cesarean in rural Rwanda

Core instructions (should be given to all women post‐cesarean)
Wound care: If you are discharged *without* wound dressings, then no wound dressings need to be used. If you are discharged *with* wound dressings, continue to use wound dressings until advised otherwise. Wound dressings should remain clean and be changed at least daily, up to the time advised by your healthcare provider to stop. Instructions on changing wound dressings, when to change dressings, and stopping the use of wound dressings will be provided. Wound dressing materials include unused sterile gauze. At any point, you can use wound dressings to prevent rubbing on the wound, but these dressings should be changed frequently (at least every day). Do not put any ointments and mixtures (traditional medicine or any other treatment, such as herbs and animal waste) on the wound unless specifically instructed by a healthcare worker.
Vaginal bleeding: Acceptable sanitary products include pads and cotton cloth to line your underwear, but do not use tampons. If you are exclusively breastfeeding, you may not see your period after delivery for 6 months when complimentary feeding begins.
Pain management: You can take medications to manage pain. The type of medication and schedule will be advised by the healthcare worker. Common medications can include: Ibuprofen, Acetaminophen, Paracetamol, or Diclofenac.
Antibiotic medications: You do not routinely need to take antibiotics after discharge. You should not take an antibiotic unless it is prescribed to you by a healthcare worker. In the limited cases where a healthcare worker prescribes antibiotics, please use exactly as prescribed.
Bowel/Bladder Concerns: Avoid holding your urine and resisting the urge to urinate. Try to urinate as often as needed. Note that bowel movements will not impact your recovery. You may notice that your bowel movements are not regular for up to 2–4 weeks after birth. To help digestion and prevent constipation, drink plenty of water and add more vegetables and whole grains (e.g. beans, lentils) to your meals.
Bathing: It is ok to bathe. If you still have gauze on the wound, remove the gauze and replace with clean and dry dressings after the bath. Make sure the water comes from a clean water source—either directly from a tap or boiled (and then cooled) before using. If you are showering, then let the water and soap run over the wound, but do not clean the wound directly. If you are using a basin to bathe, you can clean around the wound, but do not clean the wound directly. Avoid submerging the wound (swimming, using a bathtub, wading in water) for 2 weeks after your surgery. After bathing, you may pat the wound dry with a clean cloth or towel.
Clothing: Wear loose, clean clothing that will not irritate the wound. Avoid tying fabric, for example African fabric known in Rwanda as *kitenge*, on the abdomen (and specifically over the incision site) for at least 3 weeks.
Physical Activity: You are encouraged to walk regularly around and near the home for between 100 m (the length of a football pitch) to 200 m. However, you should avoid running and walking long distances, for example more than 200 m, while you heal. Minimize heavy lifting (anything heavier than the baby) and vigorous physical activity for at least 6 weeks. Specific examples: avoid digging, traveling to collect water, carrying wood for fire, and lifting older children. Abstain from sexual activity for at least 6 weeks.
Diet: Drink plenty of water. The recommended amount is at least 3 liters a day. It is ok for to eat any time after cesarean birth as long as you feel hungry. Eat small meals throughout the day, especially in the first week after surgery. Eat a variety of healthy foods and of different food groups. Avoid carbohydrate‐only (e.g. posho, rice, cassava etc.) meals and alcoholic beverages for the first few weeks. Healthy foods from different food groups include vegetables, fruits, and proteins (e.g. soya, beans).
Breastfeeding and breast care: Initiate breastfeeding immediately post‐delivery. It may take a few days for your milk to come in because you have had a cesarean delivery or heavy bleeding. During the first few days, the milk produced will be very thick and small quantities, continue to breastfeed regardless. Skin‐to‐skin, frequent feeding on breast, and hand expression will help your milk production. Speak with a health provider if you are concerned about your milk production or your baby's growth and weight gain. Cradling the base of the baby's head with your palm and supporting the baby's neck and back with your arm and the side of your body during nursing can reduce pressure on the wound. Use pillows or blankets to help support arms and baby while nursing. You may experience mild uterine cramps, especially when the baby is breastfeeding. Engorged breasts, beginning 1–2 weeks post‐delivery, will feel hard, swollen, tight, lumpy and tender to touch. Gentle massages and compressions using your finger and thumb in a C shape, moving from outwards towards the nipple, can help relieve engorged breasts. When nursing your baby, your breasts should soften. Avoid the use of any ointments or herbs on breasts.
Contraception: As you have had a cesarean birth it is even more important to allow time for your body and your womb to heal before becoming pregnant again. You should avoid becoming pregnant for at least the next 12 months. Contraception is available to prevent pregnancy; please discuss these options with your clinician before discharge, during postpartum visits and during family planning counseling. You are still able to get pregnant when your period returns while nursing, particularly if you are not exclusively breastfeeding.
Mental Health Care: The cesarean was performed for your health and/or your baby's health, and therefore you should not feel ashamed or embarrassed. Because you have undergone a cesarean, you are more at risk of having postpartum depression. Symptoms of depression involve 2 weeks of: altered sleep and appetite; loss of interest in enjoyable activities; low mood; guilt; low energy; poor concentration; moving and speaking slower than usual; intense irritability or anger; difficulty bonding or feelings of unattachment with your baby; thoughts of self‐harm or harming others. Talk with your partner, family and friends about your feelings. You can also talk with community health workers during their home visits. The coping strategies below can help improve your mental well‐being.
Coping Strategies: Connect with others. Be honest with your loved ones and let them know how you feel. Connect with other new mothers who know what you are going through; this can be an enormous help. Debrief your birthing experience with peers. Talking about the experience can be helpful to understand your emotions. Prayer and connection with local religious leaders can be helpful. Get as much rest as possible. Sleep deprivation can mimic symptoms of depression. Nap when your baby naps or put your feet up for 30 minutes of relaxation. Positive thinking: Think about the good things going on in your life. Take time for yourself each day: even if it's a short walk alone or an extra few minutes washing. You need it and deserve it. Make a list of two or three people that you can call anytime of the day or night just to talk or come over, if you are having a tough time. Keep in mind that things do get easier and you will adjust to your new life sooner than you think.
Social support at home: Seek support from family, friends or neighbors with day‐to‐day tasks and care of other children.

**TABLE 3:** Additional postpartum instructions to be communicated to specific groups of women post‐cesarean in rural Rwanda

Additional instructions (optional to be given to all post‐cesarean)	
Who should receive this instruction?	Instruction
Patients with sutures	If sutures are present, please return to the health center between 1 and 2 weeks from discharge for suture removal. The healthcare worker may give you different instructions on timing of removal, depending on the type of suture, site and condition of the wound.
Loss of infant	*Grief* is a normal reaction to loss that may include intense emotions, guilt, sadness, and appetite and sleep changes. The intensity of the reaction usually diminishes by 6 months. If grief causes you to become unable to carry out normal activities for at least 2 weeks, it is considered depression and you should seek medical care. You should avoid becoming pregnant for at least the next 6 months, ideally for the next 12 months.
Intimate partner violence	If you feel comfortable, you can talk to your healthcare provider for help and resources. Resources to help including *Isange* one‐stop services, housing options, community supports.
Previous mental health condition	There is a higher risk of postpartum depression in those who have previously had mental health problems. Please continue your previous mental health care after delivery or seek care if showing any danger signs.
Adolescents	There is a higher risk of postpartum depression in adolescents. Please contact your healthcare providers if you feel that you may need additional mental health care.
Poor access to healthcare system (no transportation or live far from healthcare system)	Contact your local community health worker for any questions and concerns if you are unable to access a clinic.

#### 3.1.1 Normal recovery versus danger signs

Women can experience a range of unique physical, psychological, and social changes during the postpartum period as they recover from cesarean birth.^6^, ^14^, ^15^, ^16^ The instructions include details to assure women that much of what they experience is within the normal range of recovery. However, the instructions emphasize timely identification of danger signs, when these changes go from normal to concerning, and distinguish the urgency with which to seek care—immediately within 24 h or urgently within 1–3 days. These danger signs were identified by TAG members based on their clinical training, professional experience, and with reference to other work on identifying postpartum danger signs, such as the POST‐BIRTH Warning Signs Education Program developed by the Association of Women's Health, Obstetric, and Neonatal Nurses.^17^

#### 3.1.2 Wound care and bathing

In typical recovery, the surgical wound is healed enough by postoperative day 3, such that a woman can be discharged without need to cover the wound with gauze for protection. For these women, the instructions focus on preventing trauma and potential infection to the wound site during bathing, dressing, and physical activities. For women discharged with wound dressings, there are specific details on changing dressings. In rural Rwanda and other SSA countries, some homes have poor water, sanitation, and hygiene (WASH) conditions, and as such, women discharged with wound dressings in Rwanda are currently advised to return to a health center to change these dressings in a sterilized setting.^13^, ^18^, ^19^, ^20^ The TAG discussed supplying home recovery kits with sterile gauze, saline washes, and soap for women to safely replace wound coverings at home to minimize the difficulty in returning to a health center but noted this intervention needed more exploration before formal recommendation.

#### 3.1.3 Medications post‐discharge

In SSA, there are high rates of post‐cesarean antibiotic use without indication.^21^ Although it is difficult to access antibiotics outside clinical settings in Rwanda, in other SSA countries, individuals can access antibiotics without prescription.^22^ Further research (Bikorimana et al, submitted for publication) and some TAG members' professional experiences also suggest that women are using oral or topical traditional medicines during their cesarean recovery.^8^, ^23^ The drafted post‐cesarean discharge instructions emphasize that women should strictly adhere to their healthcare provider's instructions on the type of medication and regimen schedule in areas of pain management, antibiotics, and wound care. Given that further research on the risks and benefits of other medications, traditional medicines, ointments, and therapies is needed, these medicines should be discussed with a healthcare provider before use.

#### 3.1.4 Personal hygiene and nutrition

During the postpartum period, good hygiene practices are essential. The TAG discussed concerns about home WASH conditions and the association of WASH conditions and risk of surgical site infections (Hakizimana et al., submitted for publication); therefore, some instructions specifically emphasize the importance of safe water sources for bathing. The instructions also provide a list of commonly available sanitary products that are safe to use and recommendations to change their pads/pieces of cloth regularly throughout the day.^1^ The instructions recommend that women maintain a nutritious diet including a variety of different food groups in order to return to/maintain normal bowel function and to support breastfeeding.^1^, ^24^, ^25^ The TAG discussed the importance of recognizing traditional and local practices around diet and personal hygiene and incorporating these into adaptations of these instructions, either emphasizing practices that facilitate recovery or pausing practices that may delay recovery.

#### 3.1.5 Breast care and breastfeeding

Promoting exclusive breastfeeding practices, as is endorsed in Rwanda, reduces risks of newborn mortality and morbidity, and improves postpartum health outcomes.^26^ Women who deliver by cesarean are more likely to delay breastfeeding initiation and should be offered additional breastfeeding and milk production counseling support at each postpartum contact.^1^, ^26^, ^27^ The instructions provide some specific tips for successful breastfeeding after cesarean. The TAG members emphasized that these may need to be modified for women who have experienced pregnancy complications or who have pre‐existing health conditions. Local practices for breast care and breastfeeding, such as special foods and local herbs to encourage milk production and nursing,^28^ could be incorporated into these instructions. Further, healthcare worker‐led and community health worker (CHW) ‐led strategies to support early and successful breastfeeding are critical both for women who deliver by cesarean or vaginally and for their infants.

#### 3.1.6 Family planning

Birth spacing is recommended for optimal outcomes for women and newborns; this is particularly important for women who give birth via cesarean to allow for full healing at the uterine surgical incision.^29^, ^30^ In Rwanda, nearly a quarter of women adopted a family planning method before discharge and an additional two‐thirds plan to engage during subsequent visits.^31^ The discharge instructions emphasize the importance of birth spacing and family planning counseling.

#### 3.1.7 Mental health care

All women are at risk of postpartum depression, and women who have delivered by cesarean in SSA are at higher risk of postpartum depression, post‐traumatic stress disorder, and postnatal emotional distress.^15^, ^16^, ^32^, ^33^ To that end, instructions related to supporting the woman's mental health was a top priority for the TAG members. However, the group also noted the limited mental health infrastructure in general care services, as is common in low‐ and middle‐income countries, and almost no mental health services integrated within maternal and child health programs.^34^ The instructions provide recommendations of coping and social support strategies to help improve and nurture mental well‐being and emphasize danger signs for when the woman should seek care in the health sector. TAG members discussed how to engage the community to improve maternal mental health during the postpartum period. Currently in Rwanda, “Expert Mothers” aid women who are new mothers, particularly for successful breastfeeding; this may be a resource to provide mental health and social support to women after cesarean but it needs further exploration before formal recommendation.^35^, ^36^

### 3.2 Follow‐up visit schedules

The timing and visit activities are provided in Tables 4 and 5. Evidence from SSA suggests that utilization at each step in the pathway of care—from antenatal care, delivery care, postpartum care, and child immunization—is a determinant of utilization in subsequent care encounters.^37^ After discussion, the TAG recommended aligning the timing and frequency of these visits with Rwanda's postpartum care framework and the WHO postnatal care guidelines to optimize service delivery with additional cesarean‐specific postpartum activities to these visits, namely: (1) at the time of discharge; (2) between 7 and 14 days after birth; and (3) 6 weeks after birth.^2^, ^38^ For women discharged with wound dressings, the visit schedule includes an additional visit at the health center within 2 days after discharge for wound dressing changes. For Rwanda, it is standard that all visits are at the local health center, which can refer women to the district hospital if a higher level of care is needed. The TAG is currently discussing opportunities for home‐based visits with a maternal and newborn health CHW for women experiencing normal recovery after cesarean.

**TABLE 4:** Postpartum care visit timeline and content for women delivering by cesarean birth in rural Rwanda

Timing	At discharge	2 days after discharge	7–14 days after delivery	6 weeks after delivery
Who should attend these visits?	All women	For patients discharged with wound dressings	All women	All women
Location	Hospital with GPs	Health center with healthcare providers; with CHW if no complications	Based on progress of recovery—Referral to hospital with GPs or health center with nurses; with CHW if no complications	Health center with nurses
Activities	Assess general well‐being and possible complications Counseling on nutrition, hygiene, family planning, and exercise Counseling on normal recovery vs danger signs Any topics patient wishes to address	Assess wound healing, replace wound dressing Assess pain level and management Assess for other complications including pregnancy and previous health complications Newborn check‐up (umbilical cord healing, breastfeeding troubleshooting, child immunization schedule) Continue discussion on family planning Any topics patient wishes to address	Assess for danger signs Check wound healing For patients with sutures, remove sutures (at hospital only) Evaluate for postpartum depression (using a validated screening tool), psychosis, and severe psychiatric illness Evaluate for signs of IPV Evaluate home conditions (if a home visit) Newborn check‐up (infant weight gain, breastfeeding troubleshooting, child immunization schedule) Continue discussion on family planning Any topics patient wishes to address	Check wound healing Assess recovery & general well‐being Preventive care (cervical smear, chronic disease management with GP Evaluate for postpartum depression and generalized anxiety disorder (using a validated screening tool), psychosis, and severe psychiatric illness Evaluate for signs of IPV and home conditions Continue discussion on family planning Child vaccination schedule Any topics patient wishes to address

Abbreviations: CHW, community health worker; GP, general practitioner; IPV, intimate partner violence.

**TABLE 5:** Visit timeline and content for postpartum care after discharge for specific groups of women delivering by cesarean birth in rural Rwanda

Population	Timing	Reasons
Loss of infant	From time of discharge continuing in all visits Adapted visitation schedule with clinical psychologist/counselors	Grief management Counseling on coping strategies and support At 4‐ to 6‐week visit, evaluate for progression of grief into depression using validated screening tool
Those with previous mental health conditions and/or experience(d) intimate partner violence	All visits Include clinical psychologist/counselors to care team during visits	If a patient does not attend her scheduled follow‐up visit, CHW or nurse will reach out to patient via phone or home visit to complete mental health check‐in Provide additional mental health support to high‐risk groups
Adolescents and/or those with poor access to health system	All visits	If a patient does not attend her scheduled follow‐up visit, CHW or nurse will reach out to patient via phone or home visit to encourage return to care

Abbreviation: CHW, community health worker.

## 4 DISCUSSION

Despite the considerable global progress in the health of women and children, the vast majority of maternal and newborn deaths occur during labor, birth, or the immediate postpartum period and are preventable.^39^, ^40^ Comprehensive and quality care during this period are vital for recovery, setting the stage for long‐term health and well‐being. Although guidelines exist for general postpartum recovery, the specific needs of women who give birth via cesarean have not been adequately addressed. This set of consensus recommendations is a first step in advancing postpartum care related to cesarean birth. Although developed specifically for women in rural Rwanda, insights reflecting professional expertise in other SSA contexts were incorporated to increase the utility of these instructions.

Two outstanding concerns regarding implementing these instructions remain. The first is in regard to the feasibility of the specific instruction recommendations for the women. In low‐resource settings, CHWs can serve as the first point‐of‐contact for primary care and linkage to the healthcare system.^41^ Studies suggest that visits by CHWs during the postnatal period improve recovery and neonatal outcomes and increase uptake of health promotion behaviors; however, a greater understanding of the factors that may influence care quality and sustainability is needed.^42^, ^43^, ^44^, ^45^ In Rwanda, a maternal and newborn CHW is integral to community‐based interventions of healthcare delivery. Within each village, the CHW provides a range of services and counseling, including conducting home‐based visits for women before and after birth, and providing referrals and accompanying women to the health center.^38^ The CHW infrastructure is valued and well‐accepted, but engaging CHWs to provide care for women after cesarean deliveries requires additional training and tools to deliver unified messages on topics specific to cesarean birth, such as wound hygiene, rest, physical activity, and mental health.^8^, ^10^ Further research on how to best integrate these instructions within CHW home visits is being explored.

Notably, there remains room for growth in the availability of and access to mental health services and psychosocial support resources across SSA.^34^ Likewise, in Rwanda, incorporating the mental health and wellness checks recommended in these instructions is constrained by the capacity of specialized mental health providers in resource‐limited settings and critical gaps in standardized screening and referral guidelines for CHWs.^46^, ^47^ In addition, how feasible these instructions are for women is determined by the availability and affordability of services and products. Specific products may vary by context, and individual preferences and values, such as type of sanitary product, pain management, and traditional medicines available. Before discharge, a healthcare provider should assess the home conditions and product availability to adapt instructions to be contextually and locally relevant for women.

Secondly, there are concerns around when and how to communicate these instructions. Although the primary end‐users of these instructions are women, the instructions are also expected to be used as a guide for health professionals and auxiliary health workers. In SSA, the hospital stay following cesarean is often 3 days after birth.^12^, ^48^ This is a prime window for visual and in‐person demonstrations, and consultations with a healthcare provider to review instructions that are clear and feasible, and any concerns are addressed. Notably, these instructions delineate between core instructions to be provided to all women, and additional instructions, that can be made available in specific circumstances. In Rwanda, mobilizing community‐level support and women's groups, such as CHWs, village leaders, and “Expert Mothers”, can complement postpartum recovery instructions and the care received at a health center.^8^, ^35^, ^36^ Lastly, while these written instructions are in English with the intention to translate into Kinyarwanda in Rwanda, the TAG discussed other formats to convey instructions, such as visual guides and infographics, and communication tools, such as mobile technology tools and video tutorials, to assist end‐users who may have limited literacy and with appropriate translation considerations.^49^, ^50^

Interestingly, the most recent WHO guidelines for antenatal care,^51^ which were released in March 2022 after the drafting of these recommendations, provide more details than previous guidelines for women who deliver via cesarean. Our recommendations here address the cesarean‐specific points in these newer guidelines, namely noting the increased anxiety women experience after cesarean delivery, the higher risk of constipation, and the need for more gradual return to physical activity when delivering via cesarean. Importantly, our recommendations expand beyond these points for other considerations that are critical and specific for women delivering via cesarean in SSA.

These consensus guidelines provide a starting point for healthcare providers and policy makers supporting women delivering via cesarean in SSA. However, they must be considered in the context of some notable limitations. First, these were developed with a focus on rural Rwanda. The TAG included members with relevant experience in other SSA countries with the goal of generalizability. When there were differing viewpoints, the group promoted the ideas most relevant for rural Rwanda and noted these instances in the narrative accompanying the recommendations. When adapting for other contexts, in‐country experts should closely review and tailor these recommendations as needed. A second limitation is that the TAG did not include women who had recently delivered via cesarean in rural Rwanda or SSA. As part of future work, we are interviewing women who have recently delivered via cesarean for input on these recommendations, including clarity, feasibility, and any outstanding concerns they had about their own recovery that were not addressed in these guidelines.

In conclusion, these consensus instructions for women at the time of discharge are a step towards providing clear guidelines to promote safe recovery after cesarean birth in rural Rwanda and other SSA contexts. We encourage discussion and more research on the feasibility of the instructions and strategies to effectively communicate them. The paucity of literature on this topic requires an interdisciplinary approach and the inclusion of providers and receivers of care. Continued collaboration among researchers, practitioners, policy makers, and women who deliver by cesarean to share their experiences in this domain furthers quality postpartum care pathways and optimization of recovery after cesarean birth with the goal of improving maternal and newborn outcomes.

## AUTHOR CONTRIBUTIONS

FK and BHG conceptualized and directed the overall objectives and the technical advisory group; FK, BHG, AL, AN, GG, SH, RLM, AAB, PK, JM, JN, RR, KM, and FS contributed to the guideline generation and interpretation; FK, BHG, and AL drafted the manuscript; AN, GG, SH, RLM, AAB, PK, JM, JN, RR, KM, and FS revised the manuscript; FK, BHG, and AL finalized the manuscript. All authors read and approved of the final manuscript.

## CONFLICT OF INTEREST

The authors declare no conflict of interest.

## Data Availability

Data sharing is not applicable to this article as no new data were created or analyzed in this study.
